# Assessing Metabolite Interactions With Chloroplastic Proteins via the PISA Assay

**DOI:** 10.21769/BioProtoc.5298

**Published:** 2025-05-05

**Authors:** Anna Karlsson, Emil Sporre, Linnéa Strandberg, Szilvia Z. Tóth, Elton P. Hudson

**Affiliations:** 1 School of Engineering Science in Chemistry, Biotechnology and Health, KTH Royal Institute of Technology, Stockholm, Sweden; 2 Institute for Integrative Biology of the Cell, Université Paris-Saclay, Paris, France; 3 Laboratory for Molecular Photobioenergetics, HUN-REN Biological Research Centre, Szeged, Hungary

**Keywords:** Chemoproteomics, PISA, Thermal proteome profiling, Protein–metabolite interaction, Plant regulation, Chloroplast isolation

## Abstract

Plants rely on metabolite regulation of proteins to control their metabolism and adapt to environmental changes, but studying these complex interaction networks remains challenging. The proteome integral solubility alteration (PISA) assay, a high-throughput chemoproteomic technique, was originally developed for mammalian systems to investigate drug targets. PISA detects changes in protein stability upon interaction with small molecules, quantified through LC–MS. Here, we present an adapted PISA protocol for *Arabidopsis thaliana* chloroplasts to identify potential protein interactions with ascorbate. Chloroplasts are extracted using a linear Percoll gradient, treated with multiple ascorbate concentrations, and subjected to heat-induced protein denaturation. Soluble proteins are extracted via ultracentrifugation, and proteome-wide stability changes are quantified using multiplexed LC–MS. We provide instructions for deconvolution of LC–MS spectra and statistical analysis using freely available software. This protocol enables unbiased screening of protein regulation by small molecules in plants without requiring prior knowledge of interaction partners, chemical probe design, or genetic modifications.

Key features

• Optimization of the PISA assay to study protein–ligand interactions in plant chloroplasts, including isolation of chloroplasts.

• Study of regulation on a proteome level, without genetic manipulation or prior knowledge of interaction partners.

• High proteome coverage, low sample requirement, 5-fold reduction of TMT-labeling cost, and short LC–MS analysis time.

• Adaptable to other organisms, such as bacteria, with minor modifications.

## Graphical overview


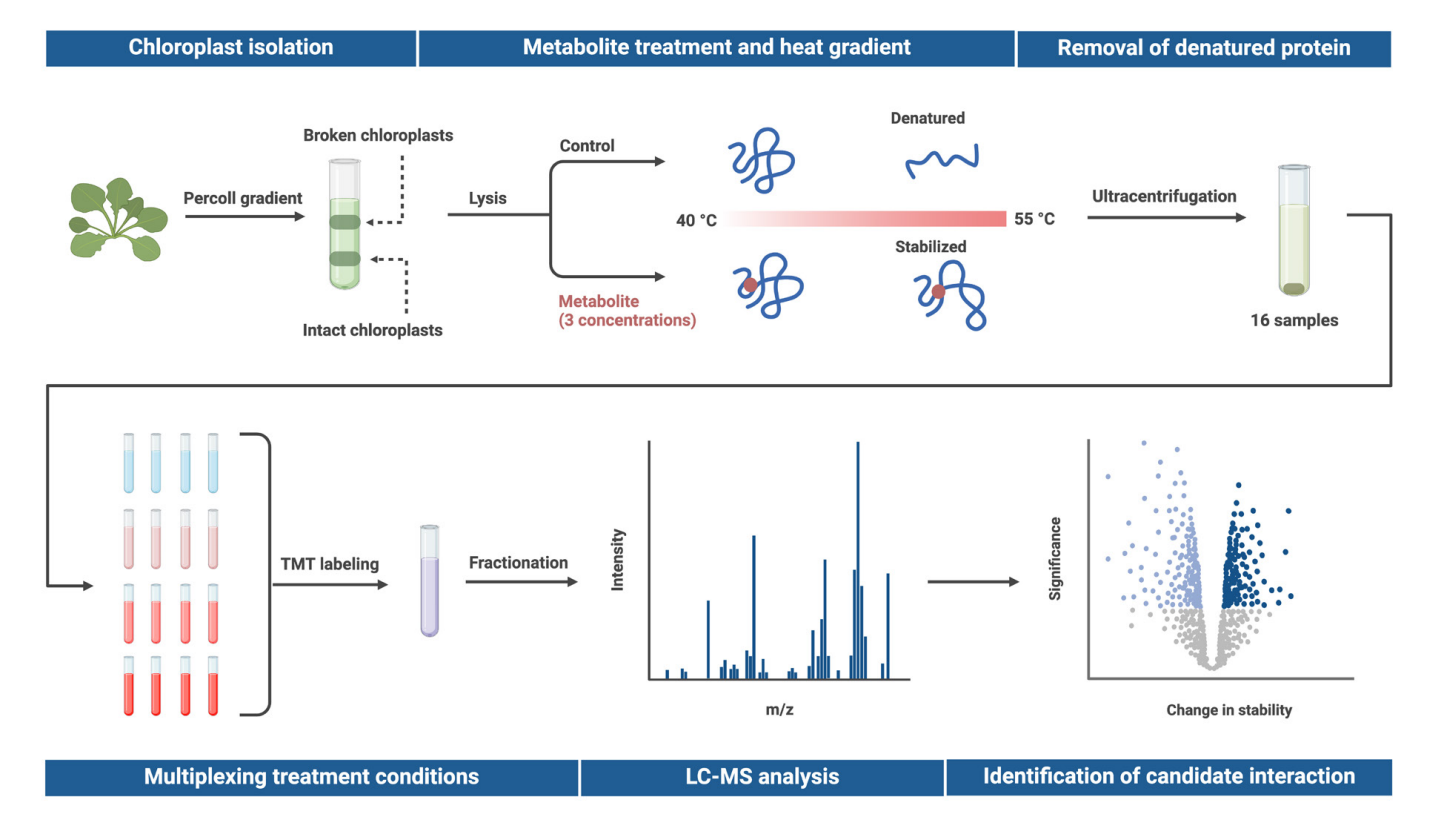


**Proteome integral solubility alteration (PISA) assay in plant chloroplasts to screen for protein–metabolite interactions.** Intact chloroplasts are isolated from leaves, lysed, and spiked with a metabolite of interest before undergoing temperature treatment. Soluble proteins are extracted via ultracentrifugation, multiplexed using tandem mass tags (TMT), and fractionated. Relative protein abundance is quantified through untargeted proteomics, and changes in protein abundance are compared to a heat-treated control sample without the metabolite. Proteins exhibiting altered temperature stability are considered potential candidates for interaction with the metabolite. To assess dose-response effects, the metabolite is tested at multiple concentrations in technical quadruplicates, with up to three concentrations analyzed using TMTpro 16plex in a single experiment. Created in BioRender. Karlsson, A. (2025) https://BioRender.com/j12o044.

## Background

Protein–metabolite interactions create dynamic regulatory networks in plants, regulating basal processes and providing robustness to changes in the environment. Understanding these regulations provides insight into agricultural and biotechnological applications but has historically been understudied due to technological limitations [1–2]. A high-throughput approach for studying protein–metabolite interactions is the proteome integral solubility alteration (PISA) assay, which detects changes in protein solubility upon metabolite binding at a proteome level using LC–MS [3]. The method is based on the principle that metabolite binding can alter protein structure and thereby temperature stability. To assess changes in protein stability, proteome extracts are subjected to a temperature gradient in the presence or absence of a metabolite. By isolating and quantifying the soluble protein fraction, the PISA assay identifies relative changes in protein solubility, indicating potential interactions with the metabolite. The protocol presented here extends the application of the PISA assay from mammalian cells to plant systems and enables the identification of metabolite-interacting proteins in the chloroplast.

The field of proteomics is rapidly advancing, with different approaches to assess protein–metabolite interactions [1–2]. Affinity purification methods use chemically modified or crosslinked metabolites as bait to capture interacting proteins. While effective for identifying high-affinity interactions, the purification process typically disrupts weaker interactions and is prone to higher false-positive rates due to the difficulty of establishing proper controls. Moreover, metabolite modifications can disrupt native interactions and require tailored probe design, limiting the number of candidate metabolites [1]. Alternatively, chemoproteomic methods, such as PISA, detect ligand-induced changes in protein properties without metabolite modification.

Thermal proteome profiling, which PISA is based on, measures protein solubility across multiple temperatures separately to fit a melting curve, determining ligand-induced thermal stability shifts [4]. However, the number of temperature points required for accurate melting curve fitting increases sample consumption and experimental complexity. In contrast, the PISA assay pools individual temperature points after heat treatment and instead quantifies the total solubility change across the temperature gradient. While it does not determine exact melting temperatures, PISA offers higher statistical power, eliminates curve-fitting bias, and significantly reduces sample requirements and mass spectrometry time [3].

In this protocol, we apply the PISA assay to assess potential interactions between chloroplastic proteins and ascorbate, a highly abundant metabolite with diverse functions in plants [5–6]. However, this method can be broadly adapted for screening protein interactions with other metabolites or small molecules, providing a versatile tool for studying plant metabolism. Additionally, the protocol can be extended to other plant compartments or bacteria with minor modifications in protein extraction and temperature gradients, further expanding its applicability to diverse biological contexts.

## Materials and reagents

**Biological materials**

1. *Arabidopsis thaliana* Col-0 (The European Arabidopsis Stock Centre, catalog number: N1093)

**Reagents**

1. 2-[4-(2-Hydroxyethyl)piperazin-1-yl]ethanesulfonic acid (HEPES) (VWR, catalog number: 30487.297)

2. Sorbitol (Merck, catalog number: S1876)

3. Magnesium chloride hexahydrate (Merck, catalog number: M9272)

4. Glycol-bis(2-aminoethylether)-N,N,N′,N′-tetraacetic acid (EGTA) (Merck, catalog number: E3889)

5. 2,2′,2″,2″′-(Ethane-1,2-diyldinitrilo)tetraacetic acid (EDTA) (Merck, catalog number: E9884)

6. Sodium bicarbonate (Merck, catalog number: S6014)

7. Magnesium sulfate heptahydrate (Merck, catalog number: 1.05886)

8. Percoll (Merck, catalog number: P7828)

9. L-Glutathione reduced (Merck, catalog number: G4251)

10. Potassium chloride (Merck, catalog number: P3911)

11. L-Ascorbic acid (Merck, catalog number: A92902)

12. 3-[4-(2-Hydroxyethyl)piperazin-1-yl]propane-1-sulfonic acid (EPPS) (Thermo Fisher Scientific, catalog number: A13714.14)

13. Dithiothreitol (Fisher Scientific, catalog number: 50-247-531)

14. Iodoacetamide (Merck, catalog number: I1149)

15. Trypsin/Lys-C protease mix (Thermo Fisher Scientific, catalog number: A40009)

16. Hydroxylamine (50% v/v) (Merck, catalog number: 438227)

17. Acetic acid (≥99%) (Merck, catalog number: A6283)

18. Formic acid (≥98%) (Merck, catalog number: 1.00264)

19. Acetonitrile (≥99.9%) (Fisher Scientific, catalog number: 15684740)

20. Trifluoroacetic acid (≥99%) (Merck, catalog number: T6399)

21. Bio-Rad Protein Assay kit (Bio-Rad, catalog number: 5000002)

23. Empore^TM^ SPE Disks for C18 StageTips (Merck, catalog number: 66883-U)

24. TMTpro^TM^ 16plex Label Reagent Set (1 × 0.5 mG) (Thermo Fisher Scientific, catalog number: A44521)

25. Pierce^TM^ High pH Reversed-Phase Peptide Fractionation kit (Thermo Scientific, catalog number: 84868)

26. NP-40 Surfact-Amps^TM^ detergent solution (Thermo Scientific, catalog number: 85125)

27. Potassium hydroxide (KOH) (Merck, catalog number: 484016)

**Solutions**

1. 1 M HEPES-KOH pH 8 buffer (see Recipes)

2. 2× concentrated isolation buffer (see Recipes)

3. HMS buffer (see Recipes)

4. Percoll gradient (see Recipes)

5. 100 mM HEPES pH 8 buffer (see Recipes)

6. 10× concentrated lysis buffer (see Recipes)

7. High 5× concentrated metabolite solution (see Recipes)

8. Medium 5× concentrated metabolite solution (see Recipes)

9. Low 5× metabolite solution (see Recipes)

10. 20 mM EPPS pH 8.5 buffer (see Recipes)

11. 200 mM dithiothreitol (see Recipes)

12. 375 mM iodoacetamide (see Recipes)

13. Trypsin/Lys-C protease mix (see Recipes)

14. 5% hydroxylamine solution (see Recipes)

15. 0.1% formic acid (see Recipes)

16. 20% formic acid (see Recipes)

17. 80% acetonitrile solution including 0.1% formic acid (see Recipes)

18. 0.1% trifluoroacetic acid (see Recipes)

19. LC-MS solvent A (see Recipes)

20. LC-MS solvent B (see Recipes)

**Recipes**

**1. 1 M HEPES pH 8 buffer**

Adjust the final solution to pH 8 using concentrated KOH. Stable for at least 6 months at room temperature.

**Table:** 

Reagent	Final concentration	Quantity or Volume
HEPES	1 M	23.8 g
MilliQ water	n/a	Add to a final volume of 100 mL
Total	n/a	100 mL

**2. 2× concentrated isolation buffer**

Prepare fresh one day before the experiment and store at 4 °C.

**Table:** 

Reagent	Final concentration	Quantity or Volume
Sorbitol	0.6 M	109.3 g
Magnesium chloride hexahydrate	10 mM	2.0 g
EGTA	10 mM	3.8 g
EDTA	10 mM	2.9 g
Sodium bicarbonate	20 mM	1.7 g
1 M HEPES pH 8 buffer (Recipe 1)	40 mM	40 mL
MilliQ water	n/a	Add to a final volume of 1 L
Total	n/a	1 L

**3. HMS buffer**

Prepare fresh one day before the experiment and store at 4 °C.

**Table:** 

Reagent	Final concentration	Quantity or Volume
Sorbitol	0.3 M	5.5 g
Magnesium sulfate heptahydrate	3 mM	74 mg
1 M HEPES pH 8 buffer (Recipe 1)	20 mM	2 mL
MilliQ water	n/a	Add to a final volume of 100 mL
Total	n/a	100 mL

**4. Percoll gradient**

Prepare fresh one day before the experiment and store at 4 °C.

**Table:** 

Reagent	Final concentration	Quantity or Volume
Percoll	50% v/v	13 mL
2× concentrated isolation buffer (Recipe 2)	50% v/v	13 mL
Glutathione	625 µM	5 mg
Total	n/a	26 mL

**5. 100 mM HEPES pH 8 buffer**

Stable for at least 6 months at room temperature.

**Table:** 

Reagent	Final concentration	Quantity or Volume
HEPES	100 mM	1 mL
MilliQ water	n/a	9 mL
Total	n/a	10 mL

**6. 10× concentrated lysis buffer**

Stable for at least 6 months at room temperature.

**Table:** 

Reagent	Final concentration	Quantity or Volume
Magnesium chloride hexahydrate	30 mM	61 g
Potassium chloride	1.5 M	1.1 g
1 M HEPES pH 8 buffer (Recipe 1)	100 mM	1 mL
MilliQ water		Add to a final volume of 10 mL
Total	n/a	10 mL

**7. High 5× concentrated metabolite solution**

Prepare quickly on ice and shielded from light to avoid degradation. Store at -20 °C in 50 μL PCR tube aliquots. Stable for at least 1 week.

**Table:** 

Reagent	Final concentration	Quantity or Volume
L-Ascorbic acid	50 mM	88 mg
1 M HEPES pH 8 buffer (Recipe 1)	100 mM	1 mL
MilliQ water		9 mL
Total	n/a	10 mL

**8. Medium 5× concentrated metabolite solution**

Prepare quickly on ice and shielded from light to avoid degradation. Store at -20 °C in 50 μL PCR tube aliquots. Stable for at least 1 week.

**Table:** 

Reagent	Final concentration	Quantity or Volume
High 5× concentrated metabolite solution (Recipe 7)	25 mM	5 mL
1 M HEPES pH 8 buffer (Recipe 1)	100 mM	0.5 mL
MilliQ water		4.5 mL
Total	n/a	10 mL

**9. Low 5× concentrated metabolite solution**

Prepare quickly on ice and shielded from light to avoid degradation. Store at -20 °C in 50 μL PCR tube aliquots. Stable for at least 1 week.

**Table:** 

Reagent	Final concentration	Quantity or Volume
Medium 5× concentrated metabolite solution (Recipe 8)	12.5 mM	5 mL
1 M HEPES pH 8 buffer (Recipe 1)	100 mM	0.5 mL
MilliQ water		4.5 mL
Total	n/a	10 mL

**10. 20 mM EPPS pH 8.5 buffer**

Adjust the final solution to pH 8.5 using concentrated KOH. Stable for at least 6 months at room temperature.

**Table:** 

Reagent	Final concentration	Quantity or Volume
EPPS	20 mM	51 mg
MilliQ water	n/a	10 mL
Total	n/a	10 mL

**11. 200 mM dithiothreitol**

Store at -20 °C in 700 μL single-use aliquots. Stable for at least 6 months.

**Table:** 

Reagent	Final concentration	Quantity or Volume
Dithiothreitol	200 mM	154 mg
MilliQ water	n/a	5 mL
Total	n/a	5 mL

**12. 375 mM iodoacetamide**

Store at -20 °C in 850 μL single-use aliquots protected from light. Stable for at least 6 months.

**Table:** 

Reagent	Final concentration	Quantity or Volume
Iodoacetamide	375 mM	328 mg
MilliQ water	n/a	5 mL
Total	n/a	5 mL

**13. Trypsin/Lys-C protease mix**

Store at -20 °C in 25 μL single-use aliquots. Stable for at least 6 months.

**Table:** 

Reagent	Final concentration	Quantity or Volume
Acetic acid (≥99%)	0.1% v/v	0.5 μL
MilliQ water	50% v/v	500 μL
Lyophilized trypsin/lysC mix	0.2 μg/μL	100 μg
Total	n/a	500 μL

**14. 5% hydroxylamine solution**

Store at 4 °C in an air-tight container for up to 1 week.

**Table:** 

Reagent	Final concentration	Quantity or Volume
Hydroxylamine (50% v/v)	5% v/v	100 μL
MilliQ water	n/a	900 μL
Total	n/a	500 μL

**15. 0.1% formic acid**

Stable at room temperature in an air-tight container for at least 6 months.

**Table:** 

Reagent	Final concentration	Quantity or Volume
Formic acid (≥98%)	0.1% v/v	50 μL
MilliQ water	n/a	50 mL
Total	n/a	50 mL

**16. 20% formic acid**

Stable at room temperature in an air-tight container for at least 6 months.

**Table:** 

Reagent	Final concentration	Quantity or Volume
Formic acid (≥98%)	20% v/v	2 mL
MilliQ water	n/a	8 mL
Total	n/a	10 mL

**17. 80% acetonitrile solution including 0.1% formic acid**

Stable at room temperature in an air-tight container for at least 1 month.

**Table:** 

Reagent	Final concentration	Quantity or Volume
Acetonitrile (≥99.9%)	80% v/v	8 mL
0.1% formic acid (Recipe 14)	20% v/v	2 mL
Total	n/a	10 mL

**18. 0.1% trifluoroacetic acid**

Stable at room temperature in an air-tight container for at least 3 months.

**Table:** 

Reagent	Final concentration	Quantity or Volume
Trifluoroacetic acid (≥99%)	0.1% v/v	50 μL
MilliQ water	99.9% v/v	Add to a final volume of 50 mL
Total	n/a	50 mL

**19. LC–MS solvent A**

Degas the solvent for 20 min in an open container using an ultrasonic cleaner. Store in an air-tight container at room temperature and prepare a new solvent every month.

**Table:** 

Reagent	Final concentration	Quantity or Volume
Acetonitrile (≥99.9%)	3% v/v	3 mL
Formic acid (>98%)	0.1% v/v	100 μL
MilliQ water	99.6% v/v	997.9 mL
Total	n/a	1 L

**20. LC–MS solvent B**

Degas the solvent for 20 min in an open container using an ultrasonic cleaner. Store in an air-tight container at room temperature and prepare a new solvent every month.

**Table:** 

Reagent	Final concentration	Quantity or Volume
Acetonitrile (≥99.9%)	95% v/v	950 mL
Formic acid (>98%)	0.1% v/v	100 μL
MilliQ water	4.9% v/v	4.9 mL
Total	n/a	1 L

**Laboratory supplies**

1. Polycarbonate bottle for Percoll gradient 50 mL (Beckman Coulter, catalog number: 357002)

2. Polycarbonate bottle for centrifugation 500 mL (Beckman Coulter, catalog number: 355664)

3. Polycarbonate tube for ultracentrifugation 1 mL (Beckman Coulter, catalog number: 343778)

4. Miracloth (Millipore, catalog number: 475855)

5. Glass beads 425–600 μm (Sigma-Aldrich, catalog number: G8772)

6. Bead beating 2 mL tubes (Sarstedt Inc, catalog number: 72.694.600)

7. Low protein binding tubes 2 mL (Sarstedt Inc, catalog number: 72.695.600)

8. Screw cap tubes 50 mL (Sarstedt, catalog number: 62.547.254)

9. Screw cap tubes 15 mL (Sarstedt, catalog number: 62.554.502)

10. PCR 8-strip tubes (BRAND, catalog number: 781320)

11. Zeba Spin desalting columns 0.5 mL (Thermo Scientific, catalog number: 89883)

12. V-bottom 96-well plate (Greiner, catalog number: 651201)

13. Clear F-bottom 96-well plate (Greiner, catalog number: 655101)

14. MS vial 1 mL (Waters, catalog number: 186000234)

15. pH indicator strips (Supelco, catalog number: 1.09535)

16. Soft paintbrush for chloroplast resuspension (Princeton Artist Brush Co., catalog number: Series 3050 P3050DF025)

17. Funnel (BRAND, catalog number: 148045)

## Equipment

1. MilliQ water purification system (Millipore, model: IQ 7000)

2. Homogenizer (IKA, model: T 25 digital ULTRA-TURRAX)

3. Absorbance plate reader (Molecular Devices, model: SpectraMax i3x)

4. Centrifuge for 2 mL tubes (Eppendorf, model: 5430 R with rotor FA-45-30-11)

5. Centrifuge for 96-well plates and 50 mL tubes (Thermo Fisher Scientific, model: Sorvall ST 16R with rotors M-20 and TX-400)

6. High-speed centrifuge for 50 and 500 mL (Beckman Coulter, model: Avanti J-26 XP with rotors JA-25.50 and JLA 8.1)

7. Ultracentrifuge (Beckman Coulter, model: Optima MAX-XP with rotor TLA 120.2)

8. Thermal cycler with gradient function (Bio-Rad, model: C1000 Touch^TM^ Thermal Cycler with Dual 48/48 Fast Reaction Module)

9. SpeedVac (Genevac, model: miVac Quatro)

10. Thermomixer (Eppendorf, model: F2.0)

11. 6500 K light source (Co/Tech, catalog number: 36-4855)

12. Bead beater (MP Biomedicals, model: FastPrep-24 5G)

13. C18 Acclaim PepMap 100 trap column (Thermo Scientific, catalog number: 164946)

14. ES802 EASY-Spray PepMap RSLC C18 column (Thermo Scientific, catalog number: 164941)

15. Nanoflow UHPLC system (Thermo Fisher Scientific, model: UltiMate 3000 RSLCnano)

16. Mass spectrometer equipped with an EASY ElectroSpray source with MS/MS resolution ≥ 45,000 (Thermo Fisher Scientific, model: Q Exactive HF Hybrid Quadrupole-Orbitrap)

17. Ultrasonic cleaner to degas LC–MS solvents (Branson, model: Bransonic CPX Digital Bath 3800)

18. Growth chamber for plants (Binder, model: KBW 400)

19. PAR light intensity meter (Skye, model: SKP 200 with sensor SKP 217)

20. Multipipette 5–50 μL (Thermo Scientific, model: Finnpipette F1 5–50 μL)

## Software and datasets

1. Q Exactive HF Tune program (Thermo Fisher Scientific, version 2.4 or later)

2. Thermo Scientific SII for Xcalibur (Thermo Fisher Scientific, version 1.6 or later)

3. MaxQuant software (Max Planck Institute of Biochemistry, version 2.2.0.0 or later)

4. SoftMax Pro plate reader software (Molecular Devices, version 6.5 or later)

5. R (R Foundation for Statistical Computing, version 4.2.2. or later)

6. Code has been deposited to GitHub: https://github.com/emilsporre/pisa (03/25/2025)

7. MS proteomics raw data used to verify the protocol [5] is available at the ProteomeXchange Consortium via the PRIDE partner repository (https://www.ebi.ac.uk/pride/) (dataset identifier: PXD052756)

## Procedure

**A. Isolation of chloroplasts**

1. Grow *A. thaliana* plants for 4–5 weeks in soil at 100 μmol photons s^-1^ m^-2^ in 12/12 h day/night cycles. Plants at this stage can be expected to have a rosette diameter of 8–12 cm.

2. The following isolation procedure is based on Aronsson and Jarvis’s protocol [7]. Because chloroplasts are fragile, it is recommended to work as quickly as possible and to work on ice or in a cold room to achieve a high yield of intact chloroplasts.

3. The day before harvest, prepare the linear Percoll gradient (see Recipes) in a 50 mL polycarbonate bottle with cap and centrifuge at 43,000× *g* for 30 min at 4 °C. Estimate one Percoll gradient tube for harvested leaves of 40 plants.

4. After preparation of the linear Percoll gradient, dilute the remaining 2× concentrated isolation buffer to 1× concentration by dilution in MilliQ water and store it at 4 °C overnight to ensure that it is cold. Estimate that you will need 1 L of 1× concentrated isolation buffer per 40 harvested plants.

5. On the day of harvest, cut leaves and place them directly into ice-cold 1× concentrated isolation buffer in a 500 mL beaker. Harvest the leaves during the last hour of the dark cycle to avoid starch build-up and avoid transferring soil to the isolation buffer, both of which can lower the yield of intact chloroplasts.

6. Fill a 50 mL beaker with 30 mL of 1× concentrated isolation buffer and add leaves up to 35 mL. Homogenize the leaves on ice using an IKA Ultra-Turrax T-25 disperser at 50% power for 3 s. Quickly move the 50 mL beaker up and down during the homogenization. Filter the solution by gravity flow through a double-layered Miracloth placed on top of a funnel and collect the filtered homogenate.

7. Repeat step A6 with the same set of leaves. The leaves should be mostly intact after the first homogenizing cycle and completely homogenized in the last cycle. Adjust the power and homogenization time if necessary (see Troubleshooting).

8. Pool the filtered homogenate into a 500 mL polycarbonate bottle with cap and centrifuge at 1,000× *g* for 5 min at 4 °C.

9. Decant the supernatant by inverting the bottle and add 2 mL of 1× concentrated isolation buffer to the pellet. Resuspend the pellet by gently swirling the bottle until the suspension appears homogenous. Optionally, facilitate the resuspension by using a clean, small, soft-bristled paintbrush to carefully loosen the pellet.

10. Transfer the solution to the Percoll gradient using a 1 mL pipette tip cut 0.5 cm from the tip. Pipette the solution slowly to the side of the Percoll gradient tube to avoid disturbing the gradient.

11. Separate intact and broken chloroplasts by centrifugation at 7,800× *g* for 10 min at 4 °C (for example, using Avanti J-26 XP with rotor JA-25.50). This will create two layers in the Percoll gradient: the upper band contains broken chloroplasts, and the lower bands contain intact chloroplasts (see Graphical overview).

12. Discard the band with broken chloroplasts by pipetting. Then, using a 1 mL pipette tip cut at the end, harvest the intact chloroplasts and place them into a 50 mL tube.

13. Wash the intact chloroplasts by gently pouring cold HMS buffer into the side of the tube up to 50 mL. Centrifuge at 1,000× *g* for 5 min at 4 °C.

14. Decant the supernatant and gently resuspend the pellet in 100–500 μL of HMS buffer by swirling or using a soft paintbrush as previously described.

15. Using a cut pipette tip, aliquot the chloroplast in 200 μL aliquots in 2 mL screw cap tubes containing 100 μL glass beads. To mimic daylight conditions, place the tubes horizontally on ice and illuminate the chloroplasts at 400 µmol photons s^-1^·m^−2^ for 5 min. Immediately snap-freeze in liquid nitrogen.

16. **Pause point:** Store chloroplasts at -80 °C until use. Chloroplasts remain stable for at least 3 months at -80 °C.

17. Optionally, save a 20 μL aliquot of chloroplast sample before snap-freezing to assess the percentage of intact chloroplasts. The percentage of intact chloroplasts can be assessed using a counting chamber or by oxygen evolution, as described in detail by Aronsson and Jarvis [7] and Joly and Carpentier [8].

**B. Protein extraction and removal of endogenous metabolites**

*Note: In Sections B and C, work on ice when possible.*

1. Thaw frozen 200 μL chloroplast aliquots on ice. For the protocol below, 1–2 aliquots are typically enough.

2. Add 17.5 μL of 10% NP-40 detergent solution to a final concentration of 0.8%.

3. Lyse the chloroplasts through three cycles of bead beating (45 s, 6.5 m/s) in a cold room. Cool the cells on ice for 30 s between cycles. Heating the protein sample at this stage can interfere with the PISA assay.

4. Centrifuge at 21,000× *g* for 5 min at 4 °C. Transfer the supernatant to a new low-bind 2 mL tube.

5. Remove endogenous metabolites using Zeba Spin desalting columns. First, place the Zeba Spin desalting columns in a low-bind 2 mL tube and remove the storage buffer by centrifuging the columns at 1,500× *g* for 1 min at 4 °C. Place the column in a new low-bind Eppendorf tube.

6. Transfer a maximum of 130 μL of cleared chloroplast lysate to the Zeba Spin desalting column and centrifuge at 1,500× *g* for 2 min at 4 °C. Collect the filtrate and discard the column. Use multiple Zeba Spin desalting columns for volumes higher than 130 μL.

7. Quantify the lysate using a protein detection assay, such as the Bio-Rad Protein Assay kit with bovine serum albumin as standard. Expect a protein concentration of 15–20 μg/μL.

**C. Heat treatment and ultracentrifugation**

1. For the following steps, 3,600 μg of protein in total is preferred when performing the experiment in quadruplicates and when using four metabolite concentrations (blank, low, medium, and high). Here, we use the ascorbate concentrations 0, 2.5, 5, and 10 mM (see Recipes). The blank condition (0 mM ascorbate) consists of 100 mM HEPES buffer pH 8.

2. Dilute the chloroplast lysate to 1.25 μg/μL in lysis buffer. For 3,600 μg of protein, this corresponds to 288 μL of 10× concentrated lysis buffer, diluted to a final volume of 2,880 μL using 100 mM HEPES buffer pH 8 and chloroplast lysate in a 15 mL tube. Add the lysate to the mixture last.

3. Split the diluted lysate into 16 replicates containing 180 μL each (4 conditions with 4 replicates per treatment) in PCR tubes.

4. Add 45 μL of 5× concentrated metabolite solution (blank, low, medium, and high) to each sample using a multipipette and incubate for 10 min. Mix by pipetting up and down 20 times.

5. During the incubation, split each sample into 16 aliquots containing 12.5 μL each in PCR tubes using a multipipette.

6. Immediately after 10 min, heat-treat all sample aliquots across a temperature range of 40–55 °C, using 1 °C intervals. Each aliquot is treated at a constant temperature, with 16 temperature points in total per replicate across the gradient. Perform the heat treatment for 3 min in a thermal cycler.

7. Allow proteins to precipitate for 6 min at room temperature after the heat treatment before placing the samples at 4 °C or on ice.

8. Add 60 μL of lysis buffer to each PCR tube, and pool the 16 heat-treated aliquots into 1 mL ultracentrifugation tubes. This results in 16 samples for the experiment (4 replicates, 4 conditions).

9. Balance the ultracentrifugation tubes to a difference of ≤10 mg using lysis buffer.

10. Ultracentrifuge at 150,000× *g* for 30 min at 4 °C.

**Critical**: The heat treatment should follow directly after the metabolite incubation and be ultracentrifuged as quickly as possible to limit metabolite metabolization and degradation of proteins. If necessary, due to a limited amount of PCR machines or ultracentrifuges, stagger the temperature and ultracentrifuge treatments with 30 min intervals. If samples are split up into different treatment rounds, it is best practice to divide replicates of different conditions equally between rounds to avoid introducing bias.

11. Carefully collect 1 mL of the supernatant from the ultracentrifuge tubes into a clean low-bind Eppendorf tube. Avoid touching the sides or bottom of the tube.

**D. Mass spectrometry workup of protein samples**

1. Add 42 μL of 200 mM dithiothreitol to a final concentration of 8 mM and reduce at 600 rpm for 45 min at 55 °C in a thermomixer.

2. Add 50 μL of 375 mM iodoacetamide to a final concentration of 17 mM and alkylate for 30 min in darkness at 600 rpm and 25 °C.

3. Quantify the protein amount using a protein detection assay, such as the Bio-Rad Protein Assay kit with bovine serum albumin as standard. Expect a protein concentration of 0.05–0.1 μg/μL.

4. Add trypsin/lys C mix in a 1:50 enzyme:protein ratio. Digest the samples overnight at 37 °C at 600 rpm.

5. Acidify the digested samples with 70 μL of 20% formic acid to pH 2 to quench the digestion and protonate peptides, enhancing their binding efficiency during C18 desalting.

6. Desalt the samples using C18 desalting StageTips. Home-made StageTips can be made by stacking six layers of C18 disks in pipette tips as described in detail by Rappsilber et al., which can be adapted to a 96-well format by stacking pipette tip racks on top of a clean V-bottom 96-well plate [9]. Alternatively, commercial C18 StageTips can be bought (such as Empore 6091 C18 StageTips). Desalting is performed with the following steps, each followed by centrifugation at 2,000× *g* for 2 min.

a. Wash the C18 StageTips with 50 μL of acetonitrile.

b. Equilibrate with 50 μL of 0.1% formic acid.

c. Load 12.5 μg of sample. If the sample volume exceeds the StageTip volume, repeat the step with the remaining sample volume until in total 12.5 μg has been loaded onto the StageTip.

d. Wash with 200 μL of 0.1% formic acid.

e. Repeat the wash.

f. Elute in 30 μL of 80% acetonitrile (v/v) solution including 0.1% formic acid (v/v). Change to a clean MS plate for the elution.

g. Repeat the elution once into the same well.

h. Dry the peptides using a SpeedVac at 45 °C.

7. **Pause point**: Store the dried peptides at -20 °C until further use. Dried peptides remain stable for at least 6 months at -20 °C.

8. Label the peptides with TMT using the following procedure:

a. Resuspend the dried peptides in 20 μL of EPPS buffer by pipetting up and down and let them sit for 20 min to dissolve the peptides.

b. While the peptides are incubating with EPPS buffer, equilibrate the TMT reagents to room temperature before opening the foil pouch.

c. Resuspend the TMT16plex 0.5 mg labels in 25 μL of anhydrous acetonitrile. Dissolve for 5 min with intermittent vortexing. Each vial is sufficient for 5 reactions and can be stored for at least 3 months at -20 °C after resuspending. For longer storage, dry the remaining TMT labeling vials before storage at -20 °C. This practice has been validated by Zecha et al. to reduce costs of TMT labeling by 5-fold [10].

d. Transfer the resuspended peptides to PCR tubes, add 5 μL of TMT label, and incubate for 1 h at room temperature at 600 rpm.

e. Quench labeling with 2 μL of 5% hydroxylamine solution.

9. Pool all samples into a 2 mL low-bind tube and adjust the volume to 1,800 μL with 0.1% trifluoroacetic acid. Verify that the pH is 2 with a pH strip and adjust with trifluoroacetic acid if needed.

10. Fractionate the peptides using the Pierce High pH Reversed-Phase Peptide Fractionation kit with the following steps:

a. Remove the protective white tip from the bottom of the column and place the column into a 2 mL tube.

b. Centrifuge at 5,000× *g* for 2 min to remove the solution and pack the resin material.

c. Remove the top screw cap and load 300 μL of acetonitrile into the column. Replace the cap, place the spin column back into a 2.0 mL sample tube, and centrifuge at 5,000× *g* for 2 min.

d. Wash the spin column twice with 0.1% trifluoroacetic acid solution, as described in the previous step.

e. Load 300 μL of the sample solution onto the column, replace the top cap, and centrifuge at 3,000× *g* for 2 min. Repeat two times to a total peptide amount of 100 μg.

f. Place the column into a new low-bind 2 mL tube. Load 300 μL of water onto the column and centrifuge at 3,000× *g* for 2 min.

g. Wash with 300 μL of 5% acetonitrile and 0.1% triethylamine (see Table 1) at 3,000× *g* for 2 min. Place the column into a new 2 mL tube.

h. Load 300 μL of the elution solution 1 according to Table 1 and centrifuge at 3,000× *g* for 2 min to collect the fraction. Repeat for the remaining elution solutions to a total of 6 fractions.

i. Transfer the collected fractions to MS vials.

11. Dry the elutions in a SpeedVac at 45 °C.

12. **Pause point**: Store dried peptides at -20 °C until further processing. Dried peptides remain stable for at least 6 months at -20 °C.

13. Resuspend each fraction in 16.7 μL of 0.1% formic acid by pipetting.

**Table 1.** Solutions used for peptide fractionation.

Solution	Acetonitrile (μL)	Triethylamine 1% (μL)*
Wash	50	950
Elution 1	150	850
Elution 2	175	825
Elution 3	200	800
Elution 4	225	775
Elution 5	250	750
Elution 6	500	500

*Part of the Pierce High pH Reversed-Phase Peptide Fractionation kit.

**E. Mass spectrometry analysis of samples**

1. Inject 4 μL of each fraction on a reversed-phase C18 nano-LC (nLC) column of a nano-LC–ESI–MS/MS instrument equipped with a C18 nano-trap column. Set the temperature of the column for nLC separation to 60 °C.

2. Separate the peptides using a flow rate of 7 μL/min using a binary solvent system consisting of LC–MS solvent A and B as specified below:

a. Equilibrate the column using 1% solvent B for 3 min.

b. Use a 120 min linear gradient increasing from 1% to 32% solvent B.

c. Wash the system by changing to 99% solvent B for 2 min and then to 1% solvent B for 1 min. Repeat 2 additional times.

d. Equilibrate the column using 1% solvent A for 6 min.

3. Quantify TMT-labeled peptides using data-dependent MS/MS acquisition in positive mode. Perform one full scan at a resolution of 120,000 at 200 mm/z with a mass range from 350 to 1,500 mm/z. Set the maximum injection time to 50 ms and the automatic gain control to 3 × 10^6^. Select the 15 most abundant peptide peaks for higher-energy collisional dissociation (HCD) fragmentation using a normalized collision energy (NCE) of 30. Perform 15 MS2 scans at a resolution of 60,000 at 200 m/z with an isolation window of 0.7 m/z. Use a maximum injection time of 120 ms and automatic gain control of 1 × 10^5^.

## Data analysis

**A. Protein quantification with MaxQuant**

1. Identify and quantify proteins using MaxQuant (or equivalent) as a search engine as follows:

a. Load the raw MS files and set fractions under the *Raw files* tab.

b. Set oxidation of methionine and N-terminal acetylation as variable modifications, and carbamidomethylation of cysteine as a fixed modification under *Group-specific parameters* → *Modifications*.

c. Specify digestion by trypsin/P and allow for two missed cleavages under *Group-specific parameters* → *Digestion*.

d. To identify TMT-labeled peptides, set quantification method to reporter ion MS2 and isobaric labeling to TMTpro 16-plex under *Group-specific parameters* → *Type*. Adjust the isotope purity using the correction matrix that comes with the TMT reagent.

e. Use the UniProt reference UP000006548 as a search library for *A. thaliana*. Load it under *Global parameters* → *Sequences.*

f. Allow for a false discovery rate of 1% under *Global parameters* → *Identification*.

2. This will output peptide and protein intensity for each TMT label (called reporter intensity). A configuration file for MaxQuant using Linux (Ubuntu) is available on GitHub (see Software and datasets). The output files evidence.txt and proteinGroups.txt in the combined output folder for the searched samples will be used for statistical analysis in Section B below. The evidence.txt file contains peptide-level data, including identified peptide-spectrum matches (PSMs) and reporter intensities for each TMT label. The proteinGroups.txt file contains protein-level data with summed TMT label intensities from peptides assigned to each protein.

**B. Statistical analysis and data interpretation**

1. Discard proteins that are not detected in n-1 of the n replicates.

2. Using the MSstats package (version 4.4.1 or later) in R [11], perform the steps below. Full R code is available at GitHub (see Software and datasets).

a. Summarize proteins and median-normalize the samples using the *proteinSummarization* function.

b. Using the function *groupComparisonTMT*, calculate the fold change of each protein treated with metabolite solution compared to the condition without metabolite. The p-values are calculated using a two-tailed Student’s t-test and corrected for multiple hypothesis testing using the Benjamini–Hochberg method.

**Figure 1.**
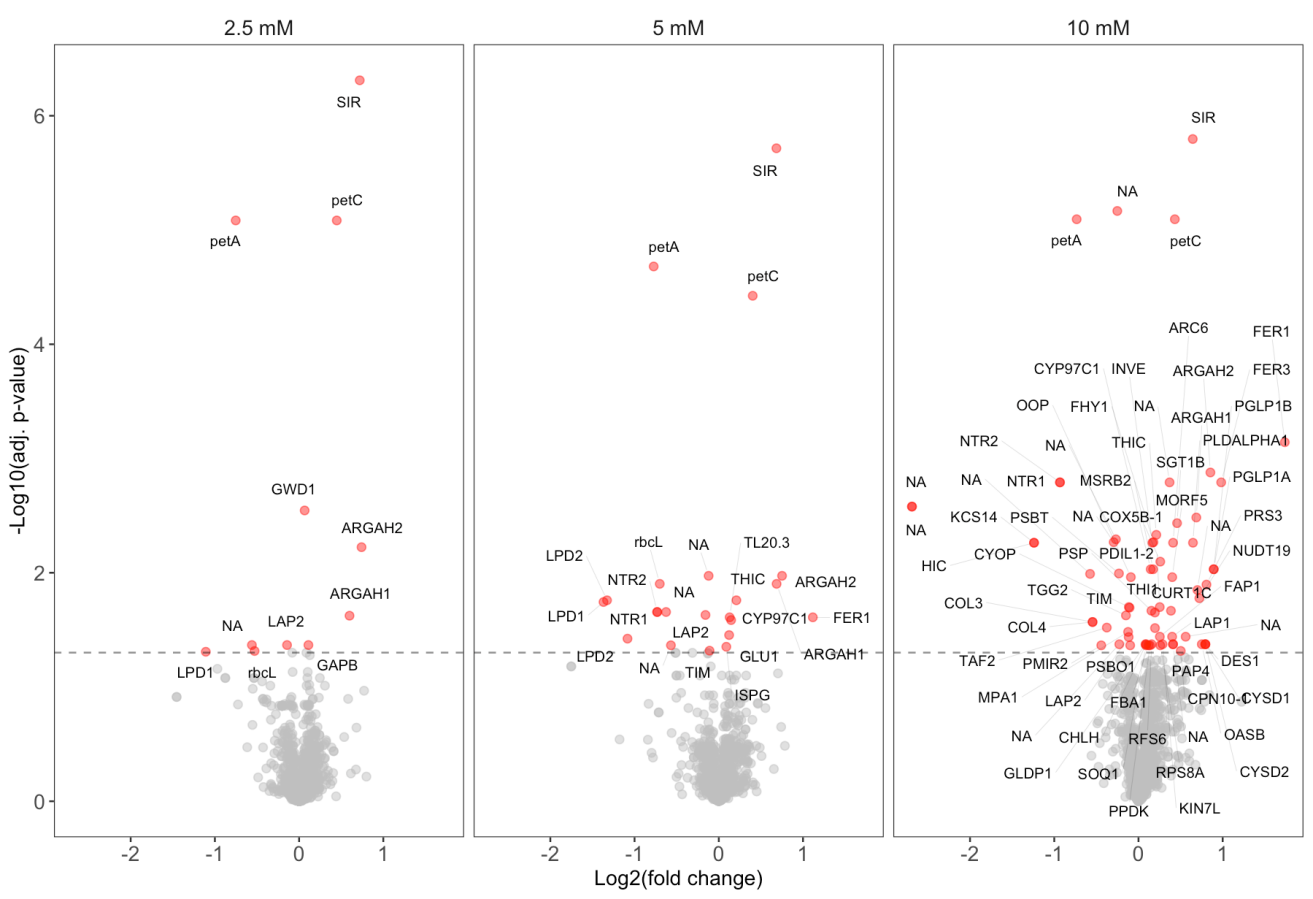
Volcano plots showing results from the PISA assay with ascorbate. Proteins with significantly altered thermal stability (adjusted p-value < 0.05) are highlighted in red for 2.5, 5, and 10 mM ascorbate, compared to the control without ascorbate. The data in this figure was originally presented and further analyzed by Tóth et al. [5].

3. Visualize proteins with affected thermal stability through volcano plots with Log2-fold change as the x-axis and -Log10 of the adjusted p-value as the y-axis (see Figure 1).

4. Potential protein–metabolite interactions are identified through low adjusted p-values and a non-zero-fold change. Proteins exhibiting consistent and significant (adjusted p-value ≤ 0.05) positive or negative fold change across multiple metabolite concentrations are more likely to represent true positives.

## Validation of protocol

This protocol has been used and validated in the following research article:

- Tóth et al. [5]. Chloroplastic ascorbate modifies plant metabolism and may act as a metabolite signal regardless of oxidative stress. Plant Physiol (Figure 6, Supplementary figure S4, Supplementary text S2, and Supplementary file 1)

## General notes and troubleshooting

**General notes**

1. When applying the PISA assay to different organisms, vary the heat temperature range to ensure optimal results. Select a gradient that captures the melting temperature of most of the proteome while keeping the gradient as narrow as possible for a higher signal-to-noise ratio [12]. The temperature stability of proteomes in common organisms has been measured by Jarzab et al. [13], and the stability of the *Arabidopsis* proteome has been measured in detail through thermal proteome profiling [14–17]. Note that the use of detergents during lysis as in this protocol can lower the melting temperature of proteins [18]. The number of peptide fractions (see step D10 of the procedure) can be fine-tuned for each species to optimize proteome coverage, particularly for large proteomes.

2. The ultracentrifugation step (see step C10 of the procedure) can be replaced by filter plates for higher throughput [19]. Separation of soluble proteins from denatured proteins can also be performed at lower speeds. While the initial thermal proteome profiling method used speeds as low as 20,000× *g*, a speed above 100,000× *g* is recommended to effectively remove denatured proteins.

**Troubleshooting**

Problem 1: Low quantity of intact chloroplasts isolated.

Possible cause: Insufficient lysis or rough handling during lysis and resuspension of pellets.

Solution: Adjust the power and time during homogenization to perform a gradual lysis of the leaves. If intact leaves are present after the last round of homogenization, increase the power by 10% and the time by 1 s in stepwise increments until no intact leaves are left. If leaves are completely homogenized early in the procedure, perform homogenization for a shorter amount of time per cycle (10% reduction in power and 1 s shorter cycle) or increase the amount of lysis buffer. Resuspend pellets using gentle agitation or a soft-bristle paintbrush. Avoid harvests of plants late in the daylight cycle to avoid starch buildup, which can make pellet resuspension difficult.

Problem 2: No clear band of intact chloroplasts on Percoll gradient separation.

Possible causes: 1) Using too much plant material for the Percoll gradient separation, which can make the bands smudged and poorly defined. 2) Not properly resuspending the pellet.

Solutions: 1) Load less material onto the Percoll gradient. 2) Check for non-resuspended lumps of plant material before loading onto the Percoll gradient.
